# Acute Middle East Respiratory Syndrome Coronavirus Infection in Livestock Dromedaries, Dubai, 2014

**DOI:** 10.3201/eid2106.150038

**Published:** 2015-06

**Authors:** Ulrich Wernery, Victor M. Corman, Emily Y.M. Wong, Alan K.L. Tsang, Doreen Muth, Susanna K. P. Lau, Kamal Khazanehdari, Florian Zirkel, Mansoor Ali, Peter Nagy, Jutka Juhasz, Renate Wernery, Sunitha Joseph, Ginu Syriac, Shyna K. Elizabeth, Nissy Annie Georgy Patteril, Patrick C. Y. Woo, Christian Drosten

**Affiliations:** Central Veterinary Research Laboratory, Dubai, United Arab Emirates (U. Wernery, K. Khazanehdari, R. Wernery, S. Joseph, G. Syriac, S.K. Elizabeth, N.A. Georgy Patteril);; University of Bonn Medical Centre, Bonn, Germany (V.M. Corman, F. Zirkel, C. Drosten); German Centre for Infection Research, Bonn (V.M. Corman, F. Zirkel, C. Drosten);; University of Hong Kong, China (E.Y.M. Wong, A.K.L. Tsang, D. Muth, S.K.P. Lau, P.C.Y. Woo);; Dubai Camel Hospital, Dubai (M. Ali);; Emirates Industry for Camel Milk and Products, Dubai (P. Nagy, J. Juhasz)

**Keywords:** MERS-CoV, Middle East respiratory syndrome coronavirus, camels, coronavirus, viruses, Dubai, livestock, dromedaries, transmission

## Abstract

Camels carry Middle East respiratory syndrome coronavirus, but little is known about infection age or prevalence. We studied >800 dromedaries of all ages and 15 mother–calf pairs. This syndrome constitutes an acute, epidemic, and time-limited infection in camels <4 years of age, particularly calves. Delayed social separation of calves might reduce human infection risk.

Middle East respiratory syndrome coronavirus (MERS-CoV) causes outbreaks and isolated cases of severe respiratory disease in humans. The virus is transmissible from human to human, but the focus of infection has remained in countries on the Arabian Peninsula. Recent reports have shown that dromedaries (*Camelus dromedarius*) across the Arabian Peninsula and parts of eastern and northern Africa have MERS-CoV antibodies (*1*–*4*). Virus detection by reverse transcription PCR (RT-PCR) and sequencing has confirmed that these antibodies are likely to be caused by infection with the same virus strains that infect humans (*5*). In singular cases, strong evidence for virus transmission between camels and humans was found (*6*,*7*). Infection of dromedaries in the laboratory has confirmed susceptibility and efficient shedding (*8*). MERS-CoV antibodies were not found in other species of livestock and leisure animals, including cattle, goats, sheep, and horses (*9*).

In the absence of a MERS-CoV vaccine, the prevention of human infections relies on knowledge of acute infection in camels. Available serologic studies indicate a high prevalence of MERS-CoV in adult camels, suggesting that MERS-CoV infection in camels may target young animals (*1*–*4*). However, only limited data on the age of animals at infection and the degree of age-specificity are available (*5*).

To best approximate the actual infectivity of virus in camels, testing should include RT-PCR and systematic virus isolation in cell culture (*10*). We recently analyzed a small group of camels in Saudi Arabia and found signs of recent acute MERS-CoV infection by demonstrating seroconversion, indicating a method for the serologic diagnosis of acute infection (*7*). To increase knowledge of acute MERS-CoV in dromedaries, we analyzed acute- and convalescent-phase MERS-CoV infections in similarly sized groups of camels of the same age in Dubai, United Arab Emirates.

## The Study

We investigated dairy, racing, and breeding dromedaries from 3 flocks on farms 20–40 km apart. When possible, blood and nasal swab specimens were obtained from all camels in the flocks during March–June 2014. Samples were grouped according to the camels’ ages rather than sampling site because livestock ages differed between sites. Serologic testing by ELISA yielded evidence of MERS-CoV antibodies in >96% of all dromedaries >2 years of age (Table 1). Seroprevalence among dromedaries <1 year of age (calves) was significantly lower but still exceeded 80%. Using cross-sectional testing, we could not discriminate between maternal and autonomous antibodies in calves. RT-PCR testing of nasal swab specimens showed a considerable prevalence of MERS-CoV RNA among all dromedaries <4 years of age but particularly in calves. Similarly, virus isolation conducted on all samples, including those RT-PCR–negative for MERS-CoV (*14*), was successful only for animals <4 years of age but particularly for calves. The prevalence of virus RNA and the rate of virus isolation were significantly higher in calves than subadults (2–4 years of age) (χ^2^, p<0.05). The higher rate of virus isolation among calves suggests increased infectivity of calves.

**Table 1:** Results of cross-sectional study of MERS-CoV antibodies and RNA and MERS-CoV isolation in dromedary camels at 3 sampling sites, Dubai, March–June, 2014*

Age group, y	Mean no. positive/no. tested (% positive)

Serum antibody detection by ELISA†	RNA detection by RT-PCR‡	Virus isolation§
Adults, >4	298/310 (96.1)	0/250 (0)	0/12 (0)
Subadults, 2–4	328/340 (96.5)	10/344 (2.9) ¶	1/14 (7.1)
Calves, <1	92/108 (85.2)¶	24/68 (35.3)¶	6/44 (13.6) ¶
Unknown	68/85 (80)	11/209 (5.3)	1/12 (8.3)
Total	786/843 (93.2)	45/871 (5.1)	8/82 (9.6)

*MERS-CoV, Middle East respiratory syndrome coronavirus; RT-PCR, reverse transcription PCR. †ELISA used a recombinant MERS-CoV globular spike (S1) domain as described by Drosten et al. (*11*), modified by the reagent manufacturer (EUROIMMUN, Lübeck, Germany) for application with camel serum as validated by Corman et al. (*12*) and Memish et al (*7*). The anti-human conjugate was replaced by an anti-camel conjugate. The test was not selective for IgG. ‡RT-PCR targeting regions upstream of the envelope gene, as described by Corman et al. (*13*). §Method as described by Drosten et al. (*14*). ¶Significantly different from grand mean (shown under Total), p<0.05 (χ^2^ test)

To understand MERS-CoV infection in dromedary calves, we investigated 24 mother–calf pairs from the breeding flock. The investigations were all conducted in May 2014. At the time of sampling, mother camels were >12–15 years of age, and calves were 4–6 months of age. As shown in Table 2, all cows were MERS-CoV antibody positive and had no signs of active MERS-CoV infection by RT-PCR and virus isolation. Of the 15 calves studied, 4 showed evidence of ongoing seroconversion during sampling days 0 and 8; on day 8, all calves were seropositive by ELISA. On sampling day 0, virus was detected in 11/15 (73.3%) calves, and on sampling day 8, it was detected in 4/15 (26.7%) calves. This overall pattern was suggestive of a recent infection peak in the flock that was already on the decline at the time of sampling. The ongoing infection in most calves suggests a general susceptibility to infection in 4- to 6-month-old dromedaries.

**Table 2:** Results of testing for the presence of MERS-CoV and MERS-CoV antibody in 15 mother–calf pairs in a dromedary breeding flock, Dubai, May 2014*

Camel	Antibody ELISA			Virus isolation			PCR, threshold cycle	
Day 0	Day 8	Day 0	Day 8	Day 0	Day 8
Mother								
M1	+	ND		−	ND		−	ND
M2	+	ND		−	ND		−	ND
M3	+	ND		−	ND		−	ND
M4	+	ND		−	ND		−	ND
M5	+	ND		−	ND		−	ND
M6	+	ND		−	ND		−	ND
M7	+	ND		−	ND		−	ND
M8	+	ND		−	ND		−	ND
M9	+	ND		−	ND		−	ND
M10	+	ND		−	ND		−	ND
M11	+	ND		−	ND		−	ND
M12	+	ND		−	ND		−	ND
M13	+	ND		−	ND		−	ND
M14	+	ND		−	ND		−	ND
M15	+	ND		−	ND		−	ND
Total	15	NA		0	NA		0	NA
Calf								
C1	±	+		+	−		19.5	−
C2	+	+		−	−		−	−
C3	+	+		−	−		24.3	−
C4	−	+		−	−		26.8	32.3
C5	±	+		−	−		30.4	−
C6	−	+		−	−		26.5	−
C7	+	+		−	−		−	−
C8	+	+		−	−		−	−
C9	+	+		+	−		23.8	−
C10	+	+		+	−		24.1	−
C11	+	+		+	−		22.3	34.2
C12	+	+		−	−		−	−
C13	+	+		−	−		22.8	34.2
C14	+	+		+	−		20.7	−
C15	+	+		−	−		32.4	35.3
Total	11 (+2)	15		5	0		11	4

*MERS-CoV, Middle East respiratory syndrome coronavirus; NA, not applicable ND, not done; +, positive; −, negative; ±, weak positive (borderline optical density range as identified by the reagent manufacturer, EUROIMMUN, Lübeck, Germany).

We sequenced genomes of 9 virus isolates, representing 3 different phylogenetic lineages, from dromedaries on the 3 farms. Phylogeny of full genomes showed that all viruses clustered according to their place of origin. The phylogenetic position of 1 of these clades suggested recent separation from viruses circulating in the eastern part of Saudi Arabia; some of the animals in the breeding flock from which the viruses were isolated had been moved temporarily to Saudi Arabia for grazing. The other clade separated from these viruses somewhat earlier, but it shared recent common ancestors with other viruses from the eastern part of the Arabian Peninsula. Samples collected in June from animals on the dairy farm yielded viruses from the same clade as that for viruses derived from different animals sampled on the same farm in March (dairy farm samples I and II) (Figure).

**Figure:**
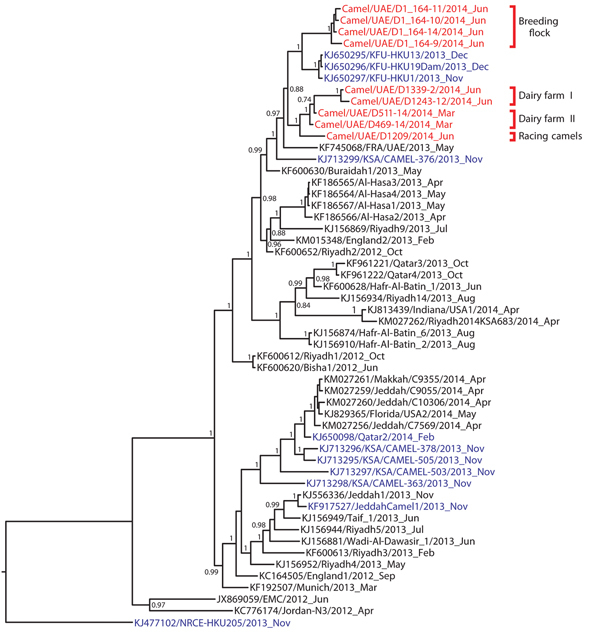
Phylogenetic analyses of the complete concatenated coding sequences of available Middle East respiratory syndrome coronavirus (MERS-CoV) genomes were done by using MrBayes v3.1 (http://mrbayes.sourceforge.net/) and a general time-reversible plus gamma distribution plus invariable site nucleotide substitution model with 2,000,000 generations sampled every 100 steps. Trees were annotated by using the last 75% of all generated trees in TreeAnnotator v.1.5 (http://beast.bio.ed.ac.uk/TreeAnnotator/) and visualized with FigTree v.1.4 (http://tree.bio.ed.ac.uk/software/figtree/). Statistical support of grouping based on Bayesian posterior probabilities is shown at all nodes (95% highest posterior density; shown if value >0.7). Red indicates the 9 camel MERS-CoV strains characterized in this study; blue indicates MERS-CoV sequences obtained from other camels. EMC, Erasmus Medical Centre; FRA, France; HKU, Hong Kong University; KFU, King Faisal University; KSA, Kingdom of Saudi Arabia; UAE, United Arab Emirates; USA, United States of America.

## Conclusions

Our findings provide evidence of infection of camel flocks in Dubai with MERS-CoV of contiguous virus clade. Similar to findings from earlier studies, we found evidence of new introductions of virus in flocks, such as the flock that temporally grazed in Saudi Arabia and was infected with a virus strain typical for Saudi Arabia (*7**,**15*). Acute MERS-CoV infection, rather than the long-term presence of virus in the dromedaries, was supported by testing mother–calf pairs. Because cows were not acutely infected before their calves, perennial persistence of MERS-CoV in adult dromedaries is unlikely. Titration and longitudinal serologic studies might have shown increases antibody titers in adult dromedaries after calves were infected. However, such studies were not possible for technical and logistical reasons, which is a clear limitation of our study.

Although we did not design our study to cover the duration of virus shedding in young dromedaries, our results suggest excretion to be short lived in individual camels. Of the 11 virus-positive calves, 5 had high virus RNA concentrations in their first samples (cycle threshold values <25) but no RNA in samples tested 8 days later. An infection experiment in adult dromedaries showed shedding occurred for <35 days after virus inoculation (*8*), which seems longer than the length of virus shedding observed for young camels in our study. However, detection sensitivity in the defined conditions of a laboratory trial might have been higher than in our study. Both studies agreed in their finding of short-lived infectivity of excreted virus: in our study, we did not detect virus in any calf on day 8, and none were detected beyond day 7 in the study by Adney et al. (*8*). Nevertheless, virus can be maintained in flocks over several weeks or months, as exemplified by the detection of the same virus clade in March and June on 1 dairy farm.

The restricted and highly compartmentalized social structure of livestock camels would provide population niches in which viruses can differentiate in isolation after bottleneck-type transmission events. This situation holds promise for control of the spread of MERS-CoV through flock management practices, and it also suggests a rather simple way of avoiding camel-to-human transmission by avoiding camels <2 years of age. Camel calves are not easily accessible by humans and instinctually avoid humans. They are generally separated from their mothers after 12 months of age (i.e., at an age when they are still likely to be infected with MERS-CoV). Humans normally come into contact with calves only after the animals have been separated from their mothers. A change in this practice (i.e., postponing separation until the calves are older) might reduce the risk for camel-to-human MERS-CoV transmission.
